# Arrêt cardiaque au cours d'une chirurgie de kyste hydatique du foie

**DOI:** 10.11604/pamj.2015.22.32.6909

**Published:** 2015-09-15

**Authors:** Omar Ouzzad, Hicham Kechna, Mohamed Karim Moudden, Khalid Chkoura, Sidi Mohamed Hanafi

**Affiliations:** 1 Pôle d'anesthésie-Réanimation-Urgences, Hôpital Militaire Moulay Ismail, Meknès, Maroc; 2 Service de Médecine Interne, Hôpital Militaire Moulay Ismail, Meknès, Maroc

**Keywords:** Choc anaphylactique, arrêt cardiaque, kyste hydatique, Anaphylactic shock, cardiac arrest, hydatid cyst

## Abstract

L'hydatidose est une pathologie fréquente qui reste encore endémique au Maroc. Sa localisation privilégiée est le foie. Son traitement repose essentiellement sur la chirurgie. Celui-ci est parfois incriminé dans la survenue de réactions allergiques sévères pouvant menacer le pronostic vital. Nous rapportons un nouveau cas de réaction anaphylactique sévère de grade IV peropératoire au cours d'une chirurgie de kystes hydatiques multiples du foie. Nous insistons sur la nécessité de sa reconnaissance rapide afin d'instaurer rapidement un traitement efficace. La prévention de cet accident est basée sur des précautions chirurgicales pour éviter les fuites ou les ruptures accidentelles peropératoires des kystes hydatiques.

## Introduction

L'hydatidose est une pathologie parasitaire causée par la forme larvaire du tænia Echinococcusgranulosus. Elle est fréquente dans les pays d’élevage comme le pourtour méditerranéen et particulièrement ceux du Maghreb. Sa localisation privilégiée est le foie. Son traitement repose essentiellement sur la chirurgie. Celui-ci peut provoquer des fuites ou des ruptures accidentelles peropératoires des kystes hydatiques; à l'origine d'une absorption systémique du liquide hydatique avec survenue de réactions allergiques sévères pouvant menacer le pronostic vital. Nous rapportons un cas d'arrêt cardiaque peropératoire au cours d'une chirurgie de kystes hydatiques multiples du foie.

## Patient et observation

Un homme âgé de 38 ans, pesant 72kg pour une taille de 173cm, déjà opéré à l’âge de 34 ans pour un kyste hydatique du foie, était programmé pour une cure chirurgicale de 3 kystes hydatiques du foie, diagnostiqué par échographie abdominale. C'est un patient sans antécédents pathologiques notables, en particulier allergiques. L'examen clinique préopératoire était normal, en dehors d'une hépatomégalie. Les examens préopératoires étaient normaux en dehors d'une sérologie hydatique positive. Le patient était classé ASA I (selon la classification de l'American Society of Anesthesiology).

La prémédication consistait en la prise de l'hydroxyzine 1mg/kg par voie orale le matin de l'intervention. Après une antibioprophylaxie par 2g d'association amoxicilline + acide clavulanique, l'anesthésie était induite par du propofol 3mg/kg, du fentanyl 3μg/kg et du bromure de rocuronium 0,5mg/kg. L'anesthésie était entretenue avec de l'isoflurane véhiculé par un mélange équimolaire d'oxygène et de protoxyde d'azote et par fentanyl en injections discontinues. Il n'y a pas eu de réinjections de curare. Le geste chirurgical devait associer aspiration du contenu kystique et résection des dômes saillants, complétés par un lavage des résidus kystiques par du sérum salé hypertonique. Quinze minutes après l'ouverture du troisième kyste (105 min après l'induction et 40min après la dernière réinjection du fentanyl), une tachycardie (136 b/mn) apparaissait, suivie d'une diminution de la pression artérielle systolique à 97, puis à 63 mm Hg, associée à une nette augmentation des pressions d'insufflation, à une désaturation en oxygène (72%), à la perception de râles sibilants au niveau des deux champs pulmonaires et apparition d'un érythème au niveau de la partie supérieure du thorax et du cou. Ce tableau était évocateur d'un choc anaphylactique avec bronchospasme. Le patient a été immédiatement mis sous 100% d'oxygène en ventilation manuelle, arrêt de l'isoflurane, simultanément, l'adrénaline à la dose de 0,2mg, l'hydrocortisone à la dose de 200mg sont administrées par voie intraveineuse, de même qu'un remplissage vasculaire rapide par du sérum salé 0,9% à la dose de 25ml/kg est démarré. Malgré ce traitement symptomatique, l’évolution était marquée par une aggravation immédiate avec la survenue d'un arrêt cardiaque. Celui-ci est récupéré après 5 minutes de massage cardiaque externe et administration intraveineuse de 1 mg d'adrénaline à deux reprises.

Après un lavage péritonéal abondant, mise en place d'une voie veineuse centrale (veine jugulaire interne droite), le patient est transporté en service de réanimation, intubé, sous ventilation mécanique. En postopératoire, il a été mis sous hydrocortisone à la dose de 100mg toutes les 6 heures, avec de l'adrénaline à la dose de 0,1μg/kg/mn en perfusion continue. L’évolution était favorable, avec levée du bronchospasme et stabilisation de l’état hémodynamique. Cela a permis d'extuber le patient après 24 heures, et de le sevrer progressivement de l'adrénaline, les corticoïdes ont été maintenues pendant 72 heures, et il a été transférer dans le service de chirurgie générale après quatre jours. Les suites étaient favorables et le patient rejoignait son domicile à j12.

## Discussion

L'hydatidose humaine est une anthropozoonose due au développement dans l'organisme de la forme larvaire du tænia Echinococcusgranulosus. Le Maghreb est considéré comme une région d'endémie hydatique. Cette parasitose atteint essentiellement le foie. Au début de son évolution, le kyste jeune se présente comme une poche liquidienne limitée par deux membranes propres. La membrane interne, dite germinative ou prolifère, donne naissance à des capsules contenant des scolex et un liquide hydatique très antigénique. Le traitement de l'hydatidose hépatique est essentiellement chirurgical, avec des techniques variées. La manipulation peropératoire du kyste expose au risque de fuites ou de ruptures accidentelles des kystes hydatiques du foie qui peuvent être à l'origine de réactions anaphylactiques secondaires à une absorption systémique du liquide hydatique [1, 2]. Les observations de Terpstra et celles de Jakubowski ont le mérite d'insister sur la rareté de ce type d'accident, sur l'absence de signes prémonitoires, et sur la difficulté du diagnostic devant un collapsus hémodynamique brutal après la stérilisation et l’évacuation du kyste [3, 4]. Le mécanisme de ces réactions est complexe. Dans certains cas, il s'agit typiquement d'une réaction d'hypersensibilité de type I liée à des immunoglobulines E en réponse à la forte concentration plasmatique des antigènes d'Ecchinococcus [5]. La réaction anaphylactique ou anaphylactoïde peut être aussi secondaire à une activation du complément avec libération d'anaphylatoxines. In vitro, le liquide hydatique active la voie alterne du complément avec formation de C3a [6]. Le diagnostic peropératoire n'est pas aisé: les signes cutanés peuvent être masqués par le drapage chirurgical ou manquer lors des réactions sévères et les manifestations cardio-respiratoires peuvent être confondues avec d'autres pathologies ayant un tableau clinique similaire.

Le choc anaphylactique doit être fortement évoqué au cours du traitement chirurgical d'un kyste hydatique devant l'installation rapide de signes cardiovasculaires (hypotension, collapsus, tachycardie ou bradycardie, voire arrêt cardiaque), de signes respiratoires (augmentation des pressions d'insufflation, bronchospasme, oedème pulmonaire), et des signes cutanés (érythème, oedème du visage). Le lien de causalité entre kyste hydatique et réaction anaphylactique peropératoire n'est retenu qu'après avoir éliminé la responsabilité des autres causes telles que les médicaments (antibiotiques, produits anesthésiques…), le latex et le produit scolicide qui peut entrainer des manifestations cardiovasculaires mimant une réaction anaphylactique.

Dans notre observation, l'hypothèse d'une réaction anaphylactique sévère de grade IV au liquide hydatique a été retenue, compte tenu que les symptômes observés sont apparus au moment de la manipulation du kyste, en dehors de tout contexte hémorragique, et à distance de l'induction anesthésique. Le choc anaphylactique est une urgence imposant une prise en charge thérapeutique immédiate. Cependant, la confirmation de la réalité du choc anaphylactique doit être apportée par un bilan biologique. Le diagnostic biologique repose sur la mesure des taux circulants des marqueurs de dégranulation des basophiles et des mastocytes (tryptase et histamine) [7]. Ce bilan n'a pas été réalisé chez notre patient en raison de sa non-disponibilité dans notre hôpital.

La stratégie de prise en chargedu choc anaphylactique a fait l'objet de plusieurs recommandations [8–10]. Le traitement du choc anaphylactique peropératoire est facilité par la mise en place préalable de la surveillance, de l′accès vasculaire et éventuellement le contrôle des voies aériennes supérieures. Le but du traitement est le rétablissement rapide des fonctions vitales perturbées par le processus d'anaphylaxie. Le retard thérapeutique est un facteur de risque de mauvais pronostic [11]. Ce traitement consiste en l'arrêt de l'exposition à l′allergène présumé, surélévation des membres inférieurs, l'arrêt de l'administration des agents anesthésiques généraux ou la diminution marquée des doses, reporter le geste chirurgical, l'interrompre ou l'accélérer avec l'accord de l’équipe chirurgicale, la ventilation avec de l'oxygène pur, le remplissage vasculaire avec des cristalloïdes et l'administration des vasopresseurs. Le vasoconstricteur de choix est l'adrénaline. Elle s′oppose aux effets systémiques induits par la libération des différents médiateurs par son action vasoconstrictrice, inotrope positive et bronchodilatatrice.Les corticoïdes sont souvent administrés à phase aiguë de choc anaphylactique, même si leurs effets sont retardés. Dans le cas rapporté, le remplissage vasculaire, l'adrénaline et les corticoïdes, n'ont pas été suffisants pour éviter l’évolution vers l'arrêt cardiaque, heureusement récupéré après augmentation des doses de l'adrénaline et massage cardiaque externe.

La prévention de ce type d'accident peropératoire repose sur l’éviction de tout passage possible de matériel hydatique dans la circulation. Elle consiste à éviter des manipulations intempestives du kyste, le vider partiellement avant l'injection du scolicide, et ne pas administrer celui-ci sous forte pression afin de ne pas créer une surdistension. La prévention par des antihistaminiques associés ou non à des corticoïdes reste controversée.

## Conclusion

La chirurgie du kyste hydatique est souvent simple, mais peut parfois se compliquer de réactions allergiques graves mettant en jeu le pronostic vital. Le diagnostic d'anaphylaxie doit être évoqué devant l'apparition soudaine d'une instabilité hémodynamique, lors de la manipulation du kyste et en dehors de tout contexte hémorragique. Le traitement adapté doit être instauré sans délai. La prévention de ces accidents est essentielle, et elle est uniquement chirurgicale.
